# A Simple Adaptive Transfer Function for Deriving the Central Blood Pressure Waveform from a Radial Blood Pressure Waveform

**DOI:** 10.1038/srep33230

**Published:** 2016-09-14

**Authors:** Mingwu Gao, William C. Rose, Barry Fetics, David A. Kass, Chen-Huan Chen, Ramakrishna Mukkamala

**Affiliations:** 1 Department of Electrical and Computer Engineering, Michigan State University, East Lansing, MI, USA; 2 Department of Kinesiology and Applied Physiology, University of Delaware, Newark, DE, USA; 3 Division of Cardiology, Department of Medicine, Johns Hopkins Medical Institutions, Baltimore, MD, USA; 4 Department of Medicine, National Yang-Ming University, Taipei City, Taiwan (R.O.C.)

## Abstract

Generalized transfer functions (GTFs) are available to compute the more relevant central blood pressure (BP) waveform from a more easily measured radial BP waveform. However, GTFs are population averages and therefore may not adapt to variations in pulse pressure (PP) amplification (ratio of radial to central PP). A simple adaptive transfer function (ATF) was developed. First, the transfer function is defined in terms of the wave travel time and reflection coefficient parameters of an arterial model. Then, the parameters are estimated from the radial BP waveform by exploiting the observation that central BP waveforms exhibit exponential diastolic decays. The ATF was assessed using the original data that helped popularize the GTF. These data included radial BP waveforms and invasive reference central BP waveforms from cardiac catheterization patients. The data were divided into low, middle, and high PP amplification groups. The ATF estimated central BP with greater accuracy than GTFs in the low PP amplification group (e.g., central systolic BP and PP root-mean-square-errors of 3.3 and 4.2 mm Hg versus 6.2 and 7.1 mm Hg; p ≤ 0.05) while showing similar accuracy in the higher PP amplification groups. The ATF may permit more accurate, non-invasive central BP monitoring in elderly and hypertensive patients.

Blood pressure (BP) waveforms become progressively distorted with increasing distance from the heart. Most notably, pulse pressure (PP) becomes increasingly amplified (see bottom of Fig. 1a). This counter-intuitive phenomenon is mainly caused by wave transmission and reflection in the arterial tree. The extent of the amplification can vary with, for example, BP- and age-induced changes in the wave travel time (which indicates the speed of the wave) and peripheral resistance-induced changes in the wave reflection coefficient (which indicates the relative magnitude of the reflected wave). So, it is BP near the heart (i.e., central BP) that directly reflects and affects cardiac performance. Further, central BP, rather than BP away from the heart (i.e., peripheral BP), is a major determinant of the degenerative changes that occur in aging and hypertension^1^. Because of its greater physiologic relevance, central BP could provide superior clinical value. However, peripheral BP waveforms are easier to measure via catheterization and applanation tonometry of a radial artery (at the wrist).

O’Rourke and co-workers previously proposed to mathematically derive the central BP waveform from a radial BP waveform^2^. They developed an average transfer function (i.e., a frequency-dependent transformation) to relate measured radial BP waveforms to measured central BP waveforms from a group of subjects and then applied the transfer function to the radial BP waveform of new subjects to predict the central BP waveform. Thereafter, some of us showed that this “generalized transfer function” (GTF) could yield good agreement with invasive central BP measurements in cardiac catheterization patients^3^,^4^. These initial, independent validation studies have received considerable attention and helped popularize the GTF.

However, since the GTF is a population average, it could often effectively assume that the PP amplification (the ratio of radial PP to central PP) is simply a fixed value. Hence, the GTF may not adapt to the aforesaid inter-subject and temporal variability in PP amplification and therefore yield nontrivial central BP errors when the PP amplification is atypical. An improved transfer function could help enhance the clinical utility of central BP, which has only been able to demonstrate marginal added clinical value over peripheral BP up to now^5^.

In this study, we conceived a new transfer function for deriving the central BP waveform from a radial BP waveform. First, the transfer function relating radial BP to central BP is defined in terms of a model of arterial wave transmission and reflection with two unknown parameters representing the wave travel time and wave reflection coefficient. Then, the parameter values of the model-based transfer function are estimated by exploiting the frequent observation that central BP waveforms exhibit exponential diastolic decays^6^,^7^,^8^,^9^. The parameter values are continually re-estimated over time for each subject. In this way, in contrast to the GTF whose underlying parameter values are population averages and therefore constant, the new transfer function is able to adapt to the arterial parameters of the subject at the time of measurement. We compared this simple “adaptive transfer function” (ATF) to multiple GTFs using the original patient data that helped popularize the GTF. Our results show that the ATF can offer significant accuracy improvements in the estimation of central BP levels in patients with low PP amplification.

## Methods

### Adaptive Transfer Function (ATF)

**Figure 1:**
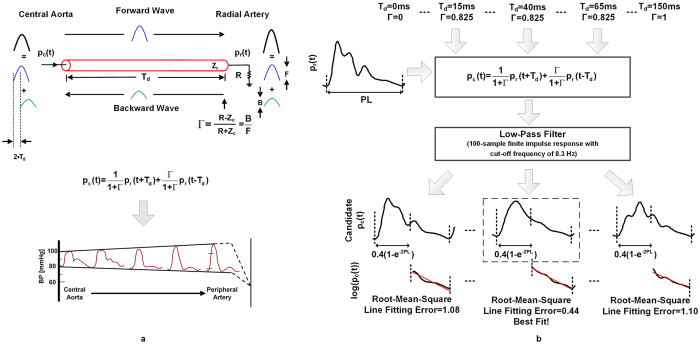
Adaptive transfer function (ATF) for deriving the central blood pressure (BP) waveform from a radial BP waveform. **(a)** Tube-load model of arterial wave transmission and reflection upon which the ATF is based (left). The model mimics the progressive amplification as well as distortion that experimental BP waveforms undergo with increasing distance from the heart (bottom) (reproduced after^23^). According to the model, the transfer function relating radial BP [p_r_(t)] to central BP [p_c_(t)] may be defined in terms of two parameters, the wave travel time [T_d_] and wave reflection coefficient [Γ]. Z_c_ is the arterial characteristic impedance, and R is peripheral resistance. **(b)** The parameters of the model-based transfer function are determined from only the radial BP waveform by exploiting the frequent observation that central BP waveforms exhibit exponential diastolic decays. In particular, the parameters, and thus the central BP waveform, are estimated so as to make the diastolic intervals of the central BP waveform as exponential as possible in the least squares sense. PL is pulse length.

The ATF transforms a radial BP waveform into the central BP waveform based on physiologic modeling and knowledge. The procedure is shown in Fig. 1 and described below.

A tube-load model is employed to represent arterial wave transmission and reflection (see top of Fig. 1a). The tube represents the wave travel path between the ascending aorta and a radial artery, while the terminal load represents the arterial bed distal to the radial artery. (Note that the wave travel path to other peripheral arteries could be represented by placing similar combinations of tubes and loads in parallel.) The tube accounts for large artery inertance [L] and compliance [C] and therefore exhibits constant characteristic impedance [Z_c_ = √(L/C)] and allows waves to travel along the entire tube with constant time delay or wave travel time [T_d_ = √(LC)]. The load accounts for the peripheral resistance [R]. While previous tube-load models have represented the load with a three-parameter Windkessel (accounting for peripheral resistance and compliance while matching the tube impedance at infinite frequency)^10^, the purely resistive load turned out to suffice here (see below).

In this model, a pressure wave travels from the tube entrance (i.e., central aorta) to the tube end/terminal load (i.e., radial artery) without distortion (Forward Wave in Fig. 1a). This wave is reflected in the opposite direction at the radial artery impedance mismatch site, with magnitude relative to the forward wave given by the constant wave reflection coefficient [Γ = (R − Z_c_)/(R + Z_c_)]. The reflected wave likewise travels along the tube without distortion (Backward Wave in Fig. 1a). The BP waveform at a given site on the tube therefore equals the sum of the forward and backward waves at that site. Since wave reflection occurs at the radial artery, there is no time delay between the forward and backward waves here. So, addition of the backward wave to the forward wave increases radial PP (see right of model in Fig. 1a). By contrast, the forward and backward waves at the central aorta are shifted by the time it takes for the wave to travel to the radial artery and back to the central aorta, which is twice the wave travel time [2·T_d_]. So, summing the backward wave with the forward wave has much less effect on central PP (see left of model in Fig. 1a). In this way, the model is able to mimic the progressive amplification as well as distortion that experimental BP waveforms undergo with increasing distance from the heart (see bottom of Fig. 1a). Note that the two parameters, T_d_ and Γ, determine the extent of the PP amplification and waveform distortion. For example, when T_d_ or Γ is small, PP amplification will be low (as there is little time delay between the forward and backward waves in the central aorta or the backward wave is small). Conversely, when the T_d_ and Γ are significant, PP amplification will be high. Further, by expressing the BP waveforms at the central aorta and radial artery in the model as the sum of appropriately time shifted forward and backward waves, a transfer function relating the radial BP waveform [p_r_(t)] to the central BP waveform [p_c_(t)] may be defined in terms of T_d_ and Γ (see transfer function equation in the time-domain in Fig. 1a). Hence, reliable estimation of these two parameters may yield a transfer function that can adapt to the prevailing PP amplification of the subject so as to more accurately estimate central BP.

The two model parameters, and thus the central BP waveform, are estimated from only the radial BP waveform (sampled at 200 Hz) by assuming that the central BP waveform exhibits exponential diastolic decays (see Fig. 1b). First, values for T_d_ in the wide range of 0 to 150 ms, with increments of 5 ms, and Γ in the physical range of 0 to 1, with increments of 0.05, are selected. Second, a candidate central BP waveform is computed by applying the time-domain model equation, equipped with the two selected parameter values, to the radial BP waveform. Third, a 100-sample finite impulse response low-pass filter is applied to further smooth the candidate waveform. Fourth, the diastolic interval of each beat of the candidate central BP waveform is approximated from the preceding pulse length (PL) according to the following formula: PL − 0.4(1-e^−2·PL^)^11^. Fifth, the candidate central BP waveform over each diastolic interval is log transformed, and a line is fitted to these data using standard linear regression. Lastly, the root-mean-square of the line fitting error is computed. These six steps are repeated for every pair of values in the T_d_ and Γ ranges to arrive at a set of candidate central BP waveforms. The T_d_ and Γ values and candidate central BP waveform that yield the minimum fitting error are chosen as the final estimates.

### Patient Data

The ATF was assessed and compared to GTFs using patient data that some of us previously collected under institutional review board approval from the Johns Hopkins Hospital and originally used for initial, independent validation of the GTF. These data are described in detail elsewhere^3^,^4^. Briefly, the data were from two cohorts of cardiac catheterization patients. The first cohort comprised 20 patients with a hemodynamic intervention to transiently change BP in 14 of the subjects^3^. The second cohort consisted of 19 patients without any intervention^4^. Each patient record included a radial artery waveform via an applanation tonometer and the reference central BP waveform via a micromanometer-tipped ascending aortic catheter. Both waveforms were about 20–60 sec in duration, sampled at 200 Hz, and low-pass filtered with a cutoff frequency of 15 Hz. Three of the interventions produced changes in central BP levels that lasted less than 10 beats. Since the ATF and GTF may require steady periods of data for their construction, the post-intervention waveforms for the corresponding patient records were excluded from the data analysis. Table 1 summarizes the patient and data characteristics.

### Data Analysis

Similar to the original, independent validation studies of the GTF^3^,^4^ the radial artery tonometer waveforms were calibrated to radial BP waveforms using the mean and diastolic levels of the reference central BP waveforms in order to focus on the transfer function itself in absence of the confounding effect of cuff BP calibration (see Discussion section). Steady periods of the radial and central BP waveforms were then selected for analysis. The BP waveforms in the first patient cohort were used to train the ATF and GTFs, while the BP waveforms in the second patient cohort were used to test the transfer functions. The roles of the first and second cohorts were then interchanged, and the training and testing procedure was repeated. In this way, the BP waveforms in both cohorts were utilized to assess the transfer functions without employing the same data for training and testing.

The ATF was trained in terms of the cutoff frequency of the post-low-pass filter and the type of load (resistor versus three-parameter Windkessel^11^). For comparison, three GTFs were also trained. The first GTF was constructed based on the autoregressive exogenous input (ARX) identification procedure outlined in the original, independent validation study^4^. This procedure was shown to be most effective amongst various approaches in that study. The second GTF was constructed based on a more straightforward ARX identification procedure. In particular, one half of each pair of radial and central BP waveforms was utilized to determine the time delay ranging from −30 to 0 samples and the ARX parameters for model orders ranging from 1 to 15 using standard least squares estimation^12^. The other half of each pair of waveforms was then employed to determine which of the 15 ARX-based transfer functions yielded the minimum average square central BP waveform estimation error^12^. The optimal transfer functions from each pair of waveforms were then averaged to arrive at the final GTF. This second GTF (GTF_ARX_) estimated central BP more accurately than the first GTF in the testing data, and increasing its model order range did not further improve the estimation (results not shown). The third GTF was built by reverse engineering the SphygmoCor device (AtCor Medical, Australia) (see Appendix). This GTF (GTF_SphygmoCor_) was a 34-sample finite impulse response filter at a sampling frequency of 128 Hz. The GTF_SphygmoCor_ was thus investigated after resampling the waveforms to 128 Hz. GTF_SphygmoCor_ yielded virtually identical central BP waveforms as the SphygmoCor device. Table 2 summarizes the investigated transfer functions.

The testing data were divided into low, middle, and high PP amplification groups of equal sizes, and the following analysis was employed per group. The ATF was applied to the entire steady period of a measured radial BP waveform (10–35 sec in duration) to estimate T_d_ and Γ and the central BP waveform, as shown in Fig. 1b. These estimates were specifically obtained by minimizing the average of the root-mean-square of the line fitting error over all the beats in the waveform period for analysis. GTF_ARX_ and GTF_SphygmoCor_ were also applied to the same waveform to estimate the central BP waveform. The central BP waveforms estimated by the three transfer functions were then quantitatively evaluated against the reference central BP waveforms in terms of the sample-to-sample (total waveform, TW), average systolic BP (SP), average PP, average augmentation index, and average ejection interval root-mean-squared-errors (RMSEs). The analyzed radial BP waveforms were likewise evaluated. All waveforms were time aligned with the reference waveforms prior to the TW RMSE calculation. The RMSEs for the ATF were then statistically compared to the RMSEs for the two GTFs and the radial BP waveform via paired t-tests of the squared-errors with Holm’s correction for the three comparisons^13^. In addition, the T_d_ and Γ estimates of the ATF were statistically compared between pairs of the three PP amplification groups via two-sample t-tests again with Holm’s correction for the three comparisons.

## Results

### ATF Training

The ATF implemented with a purely resistive load yielded central BP waveform estimates that were essentially the same as the ATF implemented with a conventional three-parameter Windkessel load in the training data (results not shown). This finding indicates that the reflection coefficient was relatively constant over the central BP frequency range here. Hence, the simpler load was selected. The post-low-pass filter cutoff frequency for the ATF was 8.4 Hz when the first patient cohort was used as the training data and 7.9 Hz when the second cohort was used as the training data. Hence, despite the use of two training datasets, the ATF could be represented with a single procedure, as shown in Fig. 1b. Note that a post-low-pass filter did not improve the central BP estimates of the GTFs.

### Central BP Accuracy

Table 3 shows the central TW, SP, and PP RMSEs for the radial BP waveform, GTF_SphygmoCor,_ GTF_ARX_, and ATF in the testing data for the low, middle, and high PP amplification groups. The average (mean ± SD) PP amplification was 1.06 ± 0.07 for the low group, 1.25 ± 0.07 for the middle group, and 1.59 ± 0.13 for the high group. The augmentation index and ejection interval RMSEs were not included in the table, because these RMSEs did not statistically differ amongst the three transfer functions.

As expected, the RMSEs for the radial BP waveform were very large but decreased substantially with decreasing PP amplification. The RMSEs for the GTF_SphygmoCor_ were lowest in the high PP amplification group, rather than the middle PP amplification group, and were highest in the low PP amplification group. Also as expected, the RMSEs for the GTF_ARX_ were low in the middle PP amplification group and higher in the low PP amplification group. However, this transfer function surprisingly yielded low RMSEs for the high PP amplification group. By contrast, the RMSEs for the ATF were comparable in all three PP amplification groups. Further, the RMSEs for the ATF were considerably lower than those for the radial BP waveform in all three groups, significantly lower than those for both GTFs in the low PP amplification group, and even lower than those for the GTF_SphygmoCor_ in the middle PP amplification group. Most notably, in the low PP amplification group, the ATF showed average RMSE reductions of 40% relative to the GTF_ARX_ and nearly 50% relative to the GTF_SphygmoCor_.

**Figure 2:**
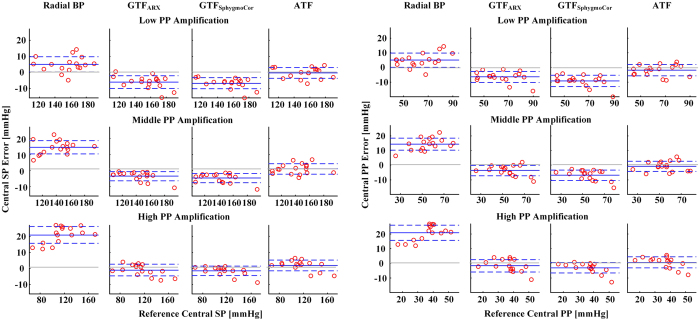
Bland-Altman plots of (a) central systolic BP (SP) errors and (b) central pulse pressure (PP) errors. The solid lines indicate the bias error, while the dashed lines indicate the limits-of-agreement. GTF_ARX_ is an effective, autoregressive exogenous input-based generalized transfer function (GTF), whereas GTF_SphygmoCor_ is a GTF that mimics the SphygmoCor device. PP amplification is the ratio of radial to central pulse pressure.

Figure 2 shows Bland-Altman plots of the central SP and PP errors in the testing data for the low, middle, and high PP amplification groups. (Note that the x-axes in the plots are the gold standard reference measurements.) These plots, which provide a visual assessment of results in Table 3, reveal that the central BP accuracy improvement afforded by the ATF was through a reduction in mainly the bias component of the RMSE.

**Figure 3:**
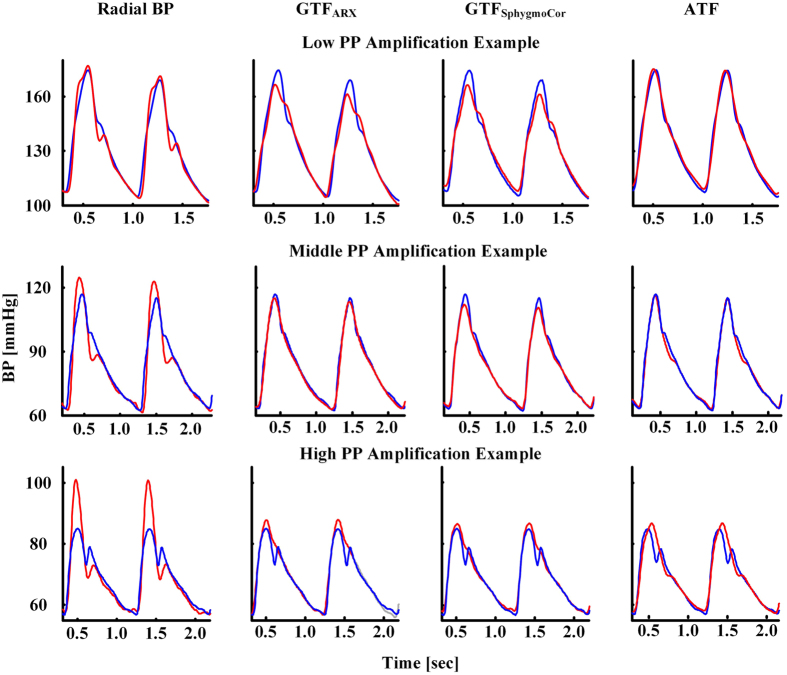
Representative examples of the estimated (red) and measured (blue) central BP waveforms.

Figure 3 shows representative examples of the estimated and measured central BP waveforms in the testing data for the low, middle, and high PP amplification groups. As can be seen, the ATF provided the best central BP waveform estimates over all three examples.

### ATF Parameter Estimates

**Figure 4:**
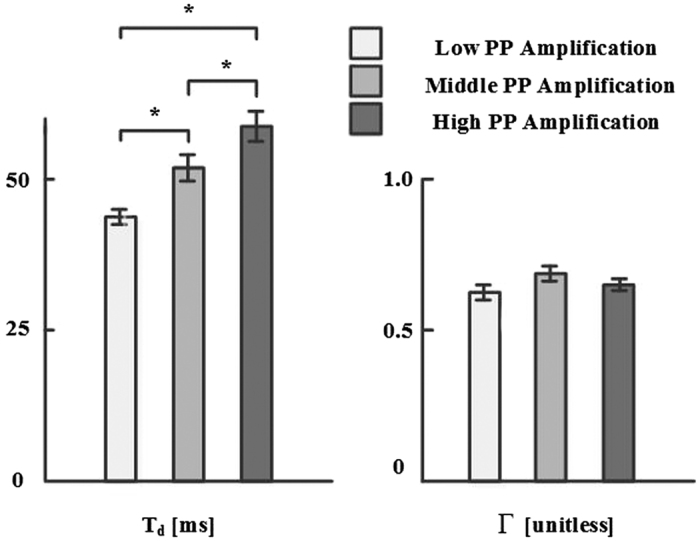
T_d_ and Γ (mean ± SE) estimates of the ATF. *Indicates statistical significance via two-sample t-tests with Holm’s correction for three comparisons.

Figure 4 shows the average T_d_ and Γ estimates of the ATF in the testing data for the low, middle, and high PP amplification groups. The T_d_ estimates significantly increased, while the Γ estimates did not change, with PP amplification. Since PP amplification can increase with T_d_ and/or Γ, these parameter estimates give further credence to the ATF.

## Discussion

We developed a simple adaptive transfer function (ATF) for mathematically deriving the central BP waveform from a radial BP waveform. The transfer function is defined in terms of the wave travel time and wave reflection coefficient parameters of a physiologic model of arterial wave transmission and reflection (see Fig. 1a). The model-based transfer function parameters are then estimated from only the radial BP waveform by assuming that the central BP waveform exhibits exponential diastolic decays (see Fig. 1b). In this way, unlike conventional GTFs, the transfer function may well adapt to the arterial properties of the subject at the time of measurement.

Over a century ago, Frank proposed that central BP waveforms could be represented with a Windkessel model, which predicts exponential diastolic decays^6^. Thereafter, exponential central BP diastolic decays have been repeatedly observed^7^,^8^,^9^. The mechanism for such decays may be as follows^14^. Forward and backward waves in the aorta have large phasic differences due to the long and varying distances between the aorta and the main reflection sites at the arterial terminations. Hence, waves with short wavelengths tend to cancel each other out in the aorta. On the other hand, waves with longer wavelengths build up in the aorta. However, these wavelengths may be long relative to the dimension of the arterial tree such that it indeed acts like a Windkessel from the perspective of the aorta. The physiologic model upon which the ATF is based captures this mechanism to a significant, but incomplete, extent^14^.

In a previous study, we proposed another ATF that employed a similar physiologic model but instead estimated the model parameters by exploiting the fact that central (ascending aortic) blood flow is negligible during diastole^15^. We also showed that this ATF could yield more accurate central BP estimates than GTFs when applied to femoral BP waveforms from animals. However, the systolic upstroke-downstroke intervals of the patient radial BP waveforms studied herein were often narrower than those of the femoral BP waveforms. As a result, our original ATF sometimes predicted central blood flow waveforms with diastolic intervals that were too wide in this study. Our conclusion is that the simple physiologic model (see Fig. 1a) may be more valid for the radial BP-to-central BP transfer function than the radial BP-to-central blood flow transfer function. We also mention that the new and simpler ATF described herein has similarity to another previously proposed ATF, which again used a similar model but estimated the model parameters by assuming that the central BP waveform was maximally smooth throughout the cardiac cycle^16^.

We assessed the ATF using the same patient data that helped popularize the GTF^3^,^4^. These data included gold standard reference central BP waveforms in addition to non-invasive radial artery tonometry waveforms from 39 cardiac catheterization patients as well as some interventions to vary BP (see Table 1). We also used these data to compare the ATF to GTFs (see Table 2). Our specific hypothesis was that changes in PP amplification (the ratio of radial PP to central PP) would adversely impact the GTFs but not the ATF. So, we divided the patient data into low, middle, and high PP amplification groups of equal sizes and studied the transfer function performance per group (see Table 3).

The GTF_SphygmoCor_, which was able to faithfully mimic the SphygmoCor device (see Appendix), estimated central BP most accurately in the high, rather than middle, PP amplification group. The reason may be that the device was trained using central and radial BP waveforms from a large number of relatively healthy subjects (but of similar average age) compared to the patients studied herein^17^. Hence, the performance of the GTF_SphygmoCor_ degraded with decreasing PP amplification and became relatively poor in the low PP amplification group.

The GTF_ARX_, which was trained using the same data and in the same way as the ATF, accurately estimated central BP in the middle PP amplification group, as expected. Its performance degraded in the low PP amplification group but was surprisingly good in the high PP amplification group. Hence, although GTFs are population averages, they have some ability to adapt to variations in PP amplification by virtue of being frequency selective.

The ATF accurately estimated central BP in all three PP amplification groups. Further, its performance was significantly better than both GTFs (see Figs 2 and 3). Indeed, the critical contribution of this study relative to previous studies of ATFs by us and others (see above and^10^) is demonstrating that an ATF can offer added value over the GTF in patients. Most notably, in the low PP amplification group, the ATF was able to reduce the central TW, SP, and PP estimation errors by an average of nearly 50% compared to the GTF_SphygmoCor_ and 40% compared to the GTF_ARX_. The low PP amplification group may not be an insignificant one. This group, by definition, constituted one-third of the patient data herein. Further, low PP amplification may occur with hypertension and aging^1^ and is caused by a short wave travel time to the radial artery and/or a small wave reflection coefficient.

The wave travel time (T_d_) estimates of the ATF indeed decreased with decreasing PP amplification, while the wave reflection coefficient (Γ) estimates did not change (see Fig. 4). However, we note that the T_d_ estimates may be more reliable, because the transfer function is often relatively insensitive to Γ. In particular, the magnitude response of the transfer function is given as follows:


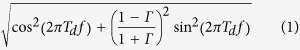


where f is frequency. Hence, the transfer function is specifically insensitive to Γ for small f (e.g., <3 Hz, which is a crucial frequency band) and moderate to high Γ (e.g., >0.4) and becomes even more insensitive to Γ with decreasing T_d_. Assuming Γ is relatively unimportant, if T_d_ is small, then the central BP waveform derived by the ATF will appear like the radial BP waveform, which nominally does not exhibit exponential diastolic decays. On the other hand, if T_d_ is large, then the derived central BP waveform will show double peaks rather than a smooth decay. Invoking the central BP exponential diastolic decay assumption may balance these two parameter settings so as to yield the proper T_d_ value. That is, if T_d_ were actually small (i.e., large pulse wave velocity), then the radial and central BP waveforms may both exhibit similar exponential diastolic decays, and the ATF would thus correctly yield a small T_d_ value. But, if T_d_ were actually large, then the radial BP waveform may not show an exponential diastolic decay, and the ATF would thus correctly yield a larger T_d_ value. In this way, the ATF was accurate over a wide range of PP amplifications.

However, the ATF was not able to offer any improvement in the estimation of the augmentation index and ejection interval of the central BP waveform (results not shown). The reason may be that its underlying physiologic model is characterized by only two parameters and is therefore too simple to account for the detailed features of the central BP waveform.

An important issue left unaddressed is practical calibration of radial artery tonometry waveforms. Like the original GTF validation studies^3^,^4^, we calibrated the radial artery tonometry waveforms using the reference central BP levels in order to focus on the transfer function. However, a major source of error in non-invasive central BP estimates is calibration with error-prone brachial BP measurements via current oscillometric cuff devices^18^,^19^. More accurate automatic cuff BP measurement methods are therefore also needed. Some of us have proposed such a method recently^20^ and hope to combine it with the simple ATF introduced herein to achieve accurate, non-invasive central BP monitoring in practice.

In conclusion, central BP is physiologically more relevant than peripheral BP and could therefore provide greater clinical value. Several studies have compared central BP and peripheral BP in terms of predicting target organ damage and cardiovascular outcomes^5^,^21^,^22^. Overall, these studies have shown that central BP does offer greater clinical value, but the improvement is not as much as one may expect. One plausible reason is nontrivial central BP measurement error. That is, in almost all of the studies, central BP was obtained by applanating a carotid artery with a tonometer, which is difficult due to surrounding loose tissue, or applying a GTF, which is a population average transformation, to a more easily measured radial BP waveform. The idea of the simple ATF introduced herein is to account for subject-specific variations in the wave travel time and wave reflection coefficient by employing physiologic modeling and knowledge and thereby improve central BP measurement accuracy. Indeed, this ATF estimated central BP levels with significantly greater accuracy than GTFs in patients with low PP amplification while showing similar accuracy in patients with higher PP amplification. Since low PP amplification can occur with high BP and aging, the ATF may provide more accurate non-invasive central BP monitoring, without compromising convenience, in hypertensive and elderly patients. Such superior accuracy could conceivably improve cardiovascular risk stratification in these important patient populations. Future studies by independent investigators may be worthwhile to confirm these results and determine if the central BP waveform as well as the wave travel time and wave reflection coefficient parameters estimated by the ATF can offer added clinical value.

## Additional Information

**How to cite this article**: Gao, M. *et al.* A Simple Adaptive Transfer Function for Deriving the Central Blood Pressure Waveform from a Radial Blood Pressure Waveform. *Sci. Rep.* **6**, 33230; doi: 10.1038/srep33230 (2016).

## Supplementary Material

Supplementary Information

## Figures and Tables

**Table 1:** Patient and data characteristics.

Patient Characteristics	Cohort 1 (n = 20)	Cohort 2 (n = 19)
Men [%]	80	74
Age [years]	59 ± 11	51 ± 16
Post Heart Transplant [%]	50	26
Coronary Artery Disease [%]	10	58
Dilated Cardiomyopathy [%]	35	0
Constrictive Pericarditis [%]	5	0
Normal [%]	0	11
Hypertension [%]	0	5
Data Characteristics (Baseline)	Cohort 1 (n = 20)	Cohort 2 (n = 19)
Central SP [mmHg]	145 ± 23	127 ± 27
Central PP [mmHg]	59 ± 15	48 ± 17
Radial PP [mmHg]	69 ± 28	52 ± 11
DP [mmHg]	86 ± 15	79 ± 18
Data Characteristics (Intervention)	Cohort 1 (n = 11)	Cohort 2 (n = 0)
Valsalva Maneuver [%]	55	—
Nitroglycerin [%]	9	—
Abdominal Compression [%]	27	—
Inferior Vena Cava [%]	9	—
|Central SP Change| [mmHg]	31 ± 20	—
|Central PP Change| [mmHg]	16 ± 11	—
|Radial PP Change| [mmHg]	14 ± 12	—
|DP Change| [mmHg]	24 ± 15	—

Values are mean ± SD or percentages. PP is pulse pressure; SP, systolic pressure, DP, diastolic pressure, |X Change|, absolute value of X change induced by intervention relative to baseline.

**Table 2:** Summary of investigated transfer functions for deriving the central blood pressure (BP) waveform from a radial BP waveform.

Transfer Function	Description	Training Data	Comments
ATF	Fig. 1	Patient Cohort 1 or 2 in Table 1	Attempts to adapt to the subject’s arterial parameters
GTF_ARX_	Data Analysis	Patient Cohort 1 or 2 in Table 1	Improved ARX-based GTF, which performed best in^3^
GTF_SphygmoCor_	Appendix	~4,000 relatively healthy subjects^16^	Perfectly mimics the SphygmoCor device

ATF is adaptive transfer function; GTF, generalized transfer function; and ARX, autoregressive exogenous input.

**Table 3:** Root-mean-squared-errors (RMSEs) between estimated and measured central BP [mmHg].

Central BP Estimates	Low PP Amplification (1.06 ± 0.07)			Middle PP Amplification (1.25 ± 0.07)			High PP Amplification (1.59 ± 0.13)		
	TW	SP	PP	TW	SP	PP	TW	SP	PP
Radial BP	6.6*	6.1#	6.1	7.8*	13.9*	13.9*	8.1*	21.6*	21.6*
GTF_SphygmoCor_	4.7*	7.5*	10.1*	3.5	5.4	7.9*	2.9	3.1	4.8
GTF_ARX_	5.2*	6.2#	7.1*	3.2	3.5	4.6	2.9	3.5	4.3
ATF	3.5	3.3	4.2	3.5	3.3	3.4	3.1	3.7	3.7

PP amplification is the ratio of radial to central pulse pressure; TW, central total waveform; SP, central systolic pressure; and PP, central pulse pressure. *and # denote statistically different (e.g., p < 0.05) or borderline statistically different (e.g., p ≈ 0.05) compared to ATF, respectively.
