# Supplementary material for: A Simple Adaptive Transfer Function for Deriving the Central Blood Pressure Waveform from a Radial Blood Pressure Waveform
Source: Sci Rep. 2016 Sep 14;6:33230. doi: 10.1038/srep33230 (PMC5021949; doi:10.1038/srep33230)
Supplement: Supplementary Information [file srep33230-s1.pdf]

A Simple Adaptive Transfer Function for Deriving the Central Blood Pressure Waveform from a Radial Blood Pressure Waveform

Mingwu Gao, William C. Rose, Barry Fetics, David A. Kass, Chen-Huan Chen, Ramakrishna Mukkamala

Appendix

The SphygmoCor device derives the central BP waveform by applying a GTF to a radial artery tonometer waveform. To determine the GTF used by the device, fifteen recordings from five subjects were analyzed. For each recording, the device generated an average radial waveform and an estimated central waveform, both sampled at 128 Hz. The two waveforms may be calibrated to BP waveforms via an arm cuff measurement.

The central BP waveform [$p_c(t)$] was assumed to be estimated from the radial BP waveform [$p_r(t)$] via a finite impulse response filter as follows:

$$p_c(t) = \sum_{k=1}^{M} h(k) p_r(t-k), \tag{1}$$

where h(k), k = 1, …, M, denotes the filter samples, and M is the number of samples or model order. MATLAB's system identification toolbox, and the `arx` function in particular, was used to determine the values of the filter samples. The model order was not known, so a variety of orders was investigated. The residual errors of the central BP waveforms estimated from the radial BP waveforms were then observed as a function of the model order. As expected, the residual error energy decreased monotonically as the model order increased. When the model order reached 34, the residual error energy dropped abruptly to essentially zero and did not change with further increases in the model order. In addition, the magnitude of the first sample of the impulse response was smaller (by a factor of three) than the next smallest sample. Therefore, models with a delay of one sample (i.e., h(1) = 0) were investigated. The residual error energy dropped sharply to a value essentially equal to zero when there were 33 or more

non-zero samples in the filter. The addition of more delay terms increased the residual error energy. Further, the addition of up to six denominator terms, which yields an infinite impulse response, did not significantly reduce the residual error energy compared to a finite impulse response. In sum, the GTF used by the Sphygmocor device is evidently a finite impulse response filter with a delay of one sample and 33 non-zero samples whose values were determined with hardly any error from pairs of BP waveforms outputted by the device.
